# Unveiling the Unspoken: Exploring Oral Manifestations of Psychological Disorders

**DOI:** 10.7759/cureus.52967

**Published:** 2024-01-25

**Authors:** Priyadharshini G, Karthikeyan Ramalingam, Pratibha Ramani

**Affiliations:** 1 Oral Pathology and Microbiology, Saveetha Dental College and Hospitals, Saveetha Institute of Medical and Technical Sciences, Saveetha University, Chennai, IND

**Keywords:** collaborative treatment, oral manifestation, quality of life, neurological, psychosomatic, mental illness, psychological, oral health, depression, stress

## Abstract

Psychological variables also interact closely with several tissues and systems leading to several diseases. The oral cavity is also linked to potential physical manifestations of psychological origin. Oral symptoms such as facial pain, oral dysaesthesia, extreme palatal erosion, or self-inflicted harm are perhaps the first or sole signs of mental health issues. It is crucial to remember that oral symptoms are frequently complex. Different people may respond differently to psychological variables and varied oral health. It is essential for dentists and medical professionals to recognize and resolve these vital problems. In this review, we have summarized the changes to oral mucosa and hard tissues and other pain disorders associated with psychological factors. Oral manifestations of a few known psychological disorders are also enumerated. This review emphasizes the role of the dentist in identifying the underlying psychological factors with oral changes. In conclusion, continuous dental care should be insisted on for patients with known mental illness to improve their quality of life. Oral health should be taken into account as part of the heightened emphasis on the overall physical well-being of those suffering from severe mental illnesses.

## Introduction and background

In medicine, oral illnesses linked to psychiatric disorders have long been recognized. The onset and development of oromucosal illnesses may be significantly influenced by emotional and psychological variables [1]. Psychosomatic conditions are characterized by physiological alterations with mixed emotional and physiological causes [2]. Numerous diseases including cardiovascular disease, hypertension, diabetes mellitus, and temporomandibular joint (TMJ) dysfunction are significantly and causally influenced by psychological and emotional aspects [3]. 

Stress being a significant role player in psychiatry has been linked to significant psychopathology, psychiatric illness, or both, as well as contributing to a great deal of mental suffering. Every physical condition has a mental component, and each person responds and deals with it differently. The oral cavity with a strong psychological potential serves as an outlet for some "instinctual" needs. Emotional and psychological factors could affect the onset and progression of oromucosal disorders [4]. The psychological changes that are associated with psychosomatic disorders are similar to those that often accompany particular emotional states, but these changes are more pronounced and long-lasting. The pathology of many diseases is significantly mediated by psychological variables, which also interact closely with several tissues and systems [5].

Mental illness is also called psychiatric illness and mental disorder. The American Psychiatric Association Diagnostic and Statistical Manual of Mental Disorders (APA DSM-5) defines a mental disorder as “a syndrome characterized by clinically significant disturbance in an individual’s cognition, emotion regulation, or behavior that reflects a dysfunction in the psychological, biological, or developmental processes underlying mental functioning”. Psychosomatic disorders are somatic illnesses caused or exacerbated by mental stress and distress. it is recognized that many diseases have psychosomatic contributors and are worsened by stress and distress. Alterations due to stress may encompass a transition in the autonomic nervous system equilibrium from parasympathetic to sympathetic control, modifications in the hypothalamic-pituitary-adrenal axis, heightened blood pressure, heart rate, and respiration, elevated blood glucose levels, augmented blood circulation to skeletal muscles, inflammation, diminished regenerative (recovery) processes, reduced digestive activity, and decreased blood flow to the prefrontal cortex during elevated levels of distress [3-5].

Thus, the oral cavity is linked to potential physical manifestations of psychological origin. Oral symptoms, such as facial pain, oral dysaesthesia, extreme palatal erosion, or self-inflicted harm, may be the first or sole signs of mental health issues. An estimated 30% of dental patients exhibit psychopathologic symptoms, which frequently go unnoticed and untreated. Similarly, poor dental health can impact other social and psychological aspects of life. In this review, the enumeration of various effects of psychological factors on the oral cavity, oral manifestations of a few known psychological disorders, and the role of dentists and mental health specialists in the management of patients with psychological issues are summarized.

Stress and oral health

Stress is defined as a physical, mental, or emotional response to events that cause bodily or mental tension [6]. Stressors and other intrinsic and extrinsic forces constantly compete for control over homeostasis, which is the basis for life. Stress cultivates physiological responses that aim to respond to the stressors, trigger an adaptive response, and return the body to its normal state. Stress causes the autonomic nervous system to release catecholamines, which then activate the serotonergic and dopaminergic systems, increase serotonin turnover, and cause the release of corticotrophin-releasing factor (CRF), glutamate, and gamma-aminobutyric acid (GABA). Corticotrophin-releasing hormone (CRH) and arginine vasopressin (AVP) are released in non-stressful conditions in a circadian, pulsatile manner, and approximately two to three secretory events each hour. These levels reach a maximum during the early morning hours while at rest, and they steadily decline throughout the day. These diurnal variations are thrown off in stressful circumstances. Acute stress increases adrenocorticotropic hormone (ACTH) and cortisol due to greater CRH and AVP pulsations [7]. The level of gonadal steroids is always changing, which could result in permanent changes to the hypothalamic-pituitary-adrenal axis that alter the amount of glucocorticoids previously generated. The main reason could be aberrant cortisol levels that harm brain cells. It has also been demonstrated that maternal stress can predispose children to react to stressful situations in later life by having higher levels of cortisol in their blood [8].

Effects of psychological factors on the oral cavity 

**Figure 1:**
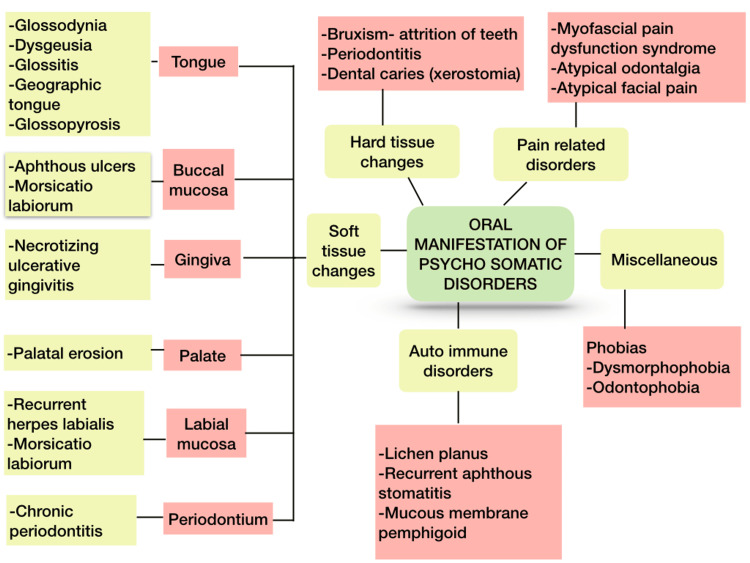
Schematic diagram shows the oral manifestation of psychosomatic disorders. Image credit: Priyadarshini, Karthikeyan Ramalingam, authors

Numerous authors have established a link between stress and high blood pressure, stomach ulcers, and type 2 diabetes. A similar study is being done to determine and support the significance of stress as an etiological factor in several oral lesions, including oral lichen planus, aphthous ulcers, burning mouth syndrome, and myo facial pain dysfunction syndrome [6]. People who are under a lot of stress may develop parafunctional habits, which are unintentional attempts to reduce stress like nail biting, chewing on objects like a pencil that causes teeth attrition, malocclusion, and tight closure of the jaws that has an impact on the masticatory muscles and cause bruxism and symptoms similar to myofascial pain dysfunction syndrome (MPDS). Vomiting brought on by gastric disorders and abnormal eating patterns might erode teeth [9]. Studies have found that the intraoral condition for patients with psychological disease was poor compared to other general patients with a high decayed-missing-filled-teeth (DMFT) index in the former compared to the latter [10]. Figure 1 shows various oral manifestations of psychosomatic disorders.

## Review

Some oral diseases associated with psychological factors

Burning Mouth Syndrome (BMS)

Pain, burning, and/or dysesthesia of the tongue and oral mucosa without pathological alterations are the hallmarks of BMS [11]. Anxiety and depression are thought to be two mental illnesses that are common and have a big impact on BMS [12]. It appears likely that both physiological and psychological variables contribute to the development, maintenance, and/or exacerbation of BMS, although it is yet unclear how these factors interact and how important each one is individually [8]. However, taste alterations and persistent burning sensation in BMS are found to be associated with large myelinated Aβ afferent damage in the trigeminal nerve or its brainstem circuits, as well as reduced Aδ fiber signaling with relatively normal C fiber function. 

Xerostomia

Hyposalivation continues to be a substantial hardship for many people, which results in xerostomia, the subjective complaint of dry mouth [13]. The relevance of psychological factors in xerostomia has been largely disregarded in studies but depressive disorders have been shown to cause decreased salivation and subjective symptoms of dry mouth [14]. Psychological problems like depression can decrease salivation by activating anticholinergic pathways. Under stress, salivary cortisol levels rise when which affects saliva output due to underactive salivary glands [13].

Recurrent Aphthous Stomatitis (RAS)

The most prevalent form of ulcerative disease of the oral mucosa, RAS, affects about 20% of the general population. RAS has been linked to psychological stress, which is a known RAS trigger that is frequently seen during stressful situations including test times for school, painful dental procedures, and major life transitions. By raising the number of leukocytes at sites of inflammation, psychological stress produces immunoregulatory activity. This is characteristically seen during the pathogenesis of RAS [15]. 

Oral Lichen Planus (OLP)

OLP is a chronic inflammatory condition characterized by white plaques or striations on both sides of the gingiva, tongue, or buccal mucosa. OLP has been connected to times of psychological stress and anxiety. In addition, they suggested that psychosocial and emotional stress may be one factor that causes reticular OLP to progress to the erosive form. Ivonavaski et al. proposed that prolonged emotional stress in OLP patients may cause psychosomatization, which in turn may contribute to the onset and clinical expression of OLP [16].

Morsicatio Labiorum and Morsicatio Buccorum

The term "morsicatio" refers to alterations in the tongue, buccal mucosa, or lip surface characteristics brought on by repeated tissue irritation by biting or sucking. It is known as "morsicatio labiorum" when this transformation occurs on the lips. Another typical type of chronic mucosal frictional keratosis, morsicatio buccorum is a condition brought on by routine buccal mucosa chewing. Both lesions are self-inflicted damage brought on by repetitive trauma, which in some cases may be linked to stress or mental instability [17]. The psychological factors include psychological conflicts and feelings including hate, jealousy, escalation, violence, a sense of helplessness and loneliness, compassion, and identity issues. Furthermore, a recent study contends that depression and morsicatio buccorum are intimately associated with one another [18]. 

Bruxism

Bruxism is characterized by teeth grinding or clenching and bracing. Studies have shown a link between bruxism and certain psychological illnesses, including hyperactivity, anxiety, behavioral disorders, issues adjusting to new situations and learning, behavioral and emotional syndromes, and stress [19]. It is widely acknowledged that stressful conditions and mental illnesses can lead to the development of occlusal parafunction and temporomandibular disorders [20].

Phantom Tooth Pain (PTP)

PTP is a deafferentation condition where the patient complains of toothache in teeth with a history of root canal therapy or in the space where teeth were located before extraction. Here the pain is protracted, persistent, and moderate to intense with little variation in intensity and is often mistaken for atypical facial pain. When the patient's symptoms can't be categorized as any physical disorder and are resistant to conventional dental and surgical treatment, and when an examination reveals high rates of psychiatric symptomatology, a psychological explanation for the diagnosis is not surprising. However, given that this hypothesis has recently been verified for other orofacial pain problems, it is still conceivable that the psychological abnormalities seen in patients with phantom pain are a result of the stress associated with the pain [21]. 

Temporomandibular Disorders (TMD)

A collection of musculoskeletal and neuromuscular disorders affecting the temporomandibular joint (TMJ), muscles of mastication, and surrounding structures are collectively referred to as TMD. TMD signs and symptoms are triggered by psychological stress; in turn, persistent signs and symptoms deteriorate the psychological state. A biopsychosocial treatment approach is necessary due to the intricate interplay between the neurobiological, psychological, and social aspects of pain [22].

Mental health disorders and their oral manifestation

**Figure 2:**
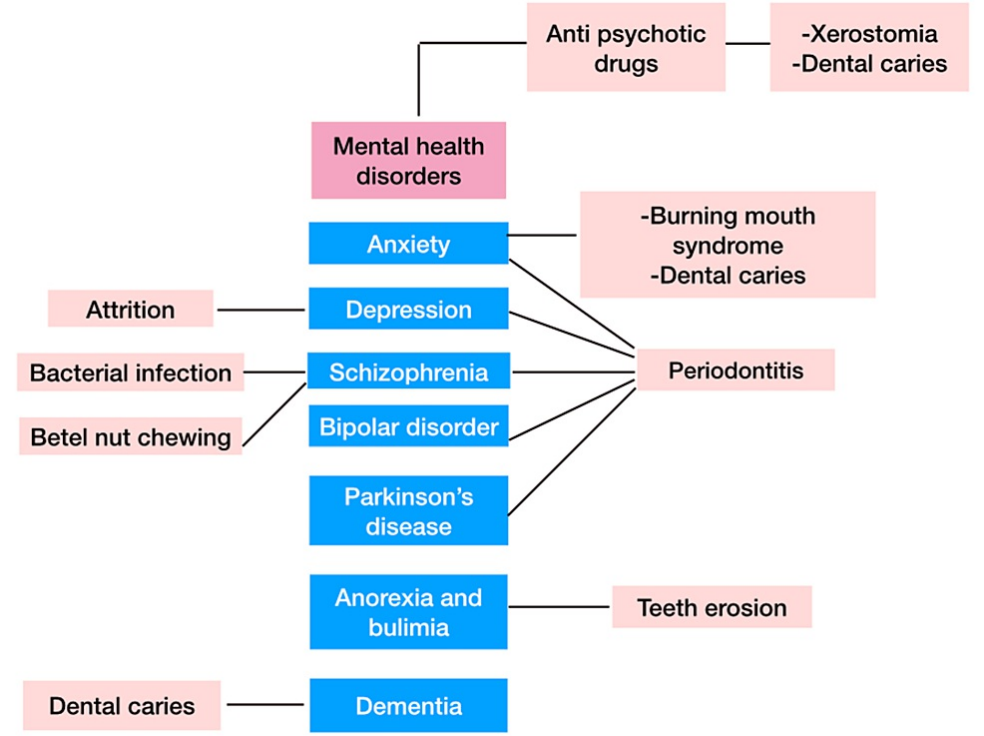
Oral manifestations of mental health disorders. Image credit: Priyadarshini, Karthikeyan Ramalingam, authors

There are psychological disorders with a known impact on oral health. Periodontitis is a common oral disease that is associated with psychological disorders like Parkinson’s disease, anxiety, depression, schizophrenia, and bipolar disorders. Dental caries, xerostomia, and bruxism are found to be associated with dementia [23,24]. Anti-psychotics and depressants may be prescribed more frequently as mental illnesses become more prevalent. These drugs carry a risk of side effects, including bruxism and xerostomia, which negatively impact the oral cavity [25]. A schematic representation of mental health disorders and their oral manifestation is illustrated in Figure 2.

Medically unexplained oral symptoms (MUOS)

The following diseases are oral conditions where the etiology of the disease has not been found but are considered to be related to psychological factors [23]. This guideline encouraged doctors to consider a diagnosis of MUOS if: (i) the patient has undergone an unusual level of investigations and/or visited a significant number of hospital specialists for their diagnosis, (ii) there is a family history of MUOS, (iii) the doctor experiences a high level of anxiety when they see a patient or their family and feel pressured to refer them for further investigations or other specialists, (iv) the doctor feels irritated with the patient or family for not getting better, (v) if the patient experiences symptoms that are out of proportion to investigations, or (vi) if a parent is overly invested in the child's condition [24].

Cases that are challenging to diagnose are frequently perceived as invisible illnesses, as there may be no apparent signs of illness upon superficial examination. Individuals grappling with such conditions grow weary of not being heard and being told that their symptoms are imagined, self-inflicted, or psychosomatic. Consequently, they often express feelings of abandonment by physicians and the healthcare system, leading to heightened risks of suicidal thoughts, suicide attempts, and suicides compared to the general population [24].

**Figure 3:**
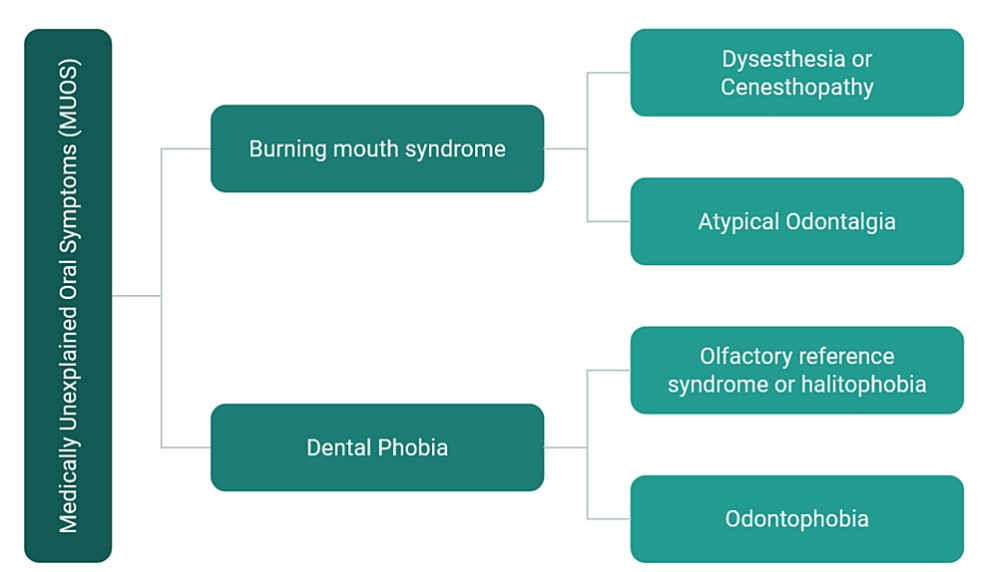
Components of medically unexplained oral symptoms (MUOS). Image credit: Priyadarshini, Karthikeyan Ramalingam, authors

These include BMS, dysesthesia or cenesthopathy, olfactory reference syndrome or halitophobia, dental phobia or odontophobia, and atypical odontalgia (Figure 3).

Neuro-oral relationship 

A link between masticatory dysfunction and cognitive decline has been found by demonstrating that the number of missing teeth is linked to an increased risk of dementia. These findings imply that tooth loss may be a risk factor for dementia and cognitive impairment [25,26]. However, since tooth loss is influenced by a lot of other factors like periodontitis, more future studies are needed to establish a relationship between brain disorders and the oral cavity. Advances in the comprehension of mind-body interactions will prompt studies into the pathophysiology of oral psychosomatic disorders and the creation of novel therapeutic strategies. Enhancing education on the intersection of medicine and psychiatry, along with the corresponding diagnostic terminology, and incorporating clinical judgment and comprehensive assessment with humility can enhance diagnostic precision and foster patient confidence.

Collaborative treatment approach 

Due to specialization and fragmentation within healthcare systems, numerous healthcare providers lack proficiency in both psychiatry and general medicine. Consequently, there exists a gap in understanding the interface between mental and somatic disorders that falls between the domains of psychiatry and medicine. Addressing diseases that involve the interaction between the brain and body poses distinct challenges. Considering the individual uniqueness, biological variations, complexities of certain illnesses, and differences in treatment tolerance, safety, and efficacy among individuals, adhering strictly to treatment guidelines derived from group data and applying them without exercising clinical judgment may not give full benefits to the patient. These numerous factors contribute to patients with complex illnesses feeling disoriented and neglected within the healthcare system.

The Perceived Stress Scale (PSS) is the most commonly used tool for measuring subjectively experienced stress. It is still a preferred option for assisting the comprehension of how various circumstances impact emotions and sense of stress. However, PSS has been found to have a unidimensional structure. Other reports indicate two-factor or three-factor structures for the same scale, and the implications must be explored [27]. Recent studies have found that stress-related biomarkers are physiological indicators of physiological processes that can be objectively tested and are either markers of the process or involved in the pathway from stress to disease. Heat shock proteins (HSPs), oxidative stress markers, innate immune markers like acute phase proteins (APPs), and chemicals released in the saliva and urine are examples of potential stress markers [28]. Kisley et al. have performed a systematic review with meta-analysis regarding poor oral health and severe mental illness. They have identified an increased prevalence of dental caries and a higher risk of total tooth loss [29].

It's crucial to remember that oral symptoms are frequently complex and that different people may respond differently to psychological variables and oral health. In recognizing and resolving these problems, dentists and other medical professionals are essential. Dentists should keep in mind any oral signs and symptoms related to psychological disorders and identify the potential psychological cause of a dental issue. Given that some patients are reluctant to seek psychiatric care, a dentist may be the first medical professional to suspect an underlying psychological disorder. For instance, anxious patients exhibit symptoms like agitation and trembling and typically ask a lot of questions regarding the procedures.

Similarly, greater emphasis on the physical well-being of individuals with severe mental illness needs to be extended to their oral health. Using common checklists to assess oral health, oral hygiene guidance, treating iatrogenic dry mouth, and early dental referrals are a few examples of potential interventions by medical professionals or mental health specialists. Collaboration is often required between dentists and mental health specialists. A full assessment and suitable counsel are best obtained by consulting with both a dentist and a mental health expert if a patient has specific questions or concerns regarding dental health and its relationship to psychological well-being. Dental care for these persons is challenging due to their lack of enthusiasm and indifference, fear of treatment, insufficient communication skills, and financial considerations [30,31].

## Conclusions

The population that is most susceptible to oral ailments has traditionally been those with serious mental disorders. This may be because of a variety of factors, including the nature of psychological illness, lack of awareness of one's oral health issues, dental phobia, and neglected oral hygiene. To enhance these patients' quality of life, it is necessary to insist on receiving the best dental treatment possible. Furthermore, the dentist plays a critical role in determining if a patient has an underlying psychiatric problem through their oral manifestations. In conclusion, there should be a greater focus on the oral health of individuals experiencing severe mental illness, and this contributes to the enhanced physical wellbeing of such individuals.
